# Shaping the Structure and Properties of TiO_2_-ZnO Oxide Coatings Produced by Plasma Electrolytic Oxidation on Titanium Substrate

**DOI:** 10.3390/ma16237400

**Published:** 2023-11-28

**Authors:** Magdalena Marny, Maciej Sowa, Alicja Kazek-Kęsik, Krzysztof Rokosz, Steinar Raaen, Patrick Chapon, Roman Viter, Roman Pshenychnyi, Wojciech Simka, Joanna Michalska

**Affiliations:** 1 Faculty of Chemistry, Silesian University of Technology, 44-100 Gliwice, Poland; magdalena.marny@polsl.pl (M.M.); maciej.sowa@polsl.pl (M.S.); alicja.kazek-kesik@polsl.pl (A.K.-K.); 2 Faculty of Electronics and Computer Science, Koszalin University of Technology, 75-620 Koszalin, Poland; krzysztof.rokosz@tu.koszalin.pl; 3 Department of Physics, Norwegian University of Science and Technology (NTNU), NO 7491 Trondheim, Norway; steinar.raaen@ntnu.no; 4 HORIBA Scientific, 14 Boulevard Thomas Gobert, Pass. Jobin-Yvon, 91120 Palaiseau, France; patrick.chapon@horiba.com; 5 Institute of Chemical Physics, Institute of Atomic Physics and Spectroscopy, University of Latvia, 1586 Riga, Latvia; roman.viter@lu.lv; 6 Medical Institute, Sumy State University, 40018 Sumy, Ukraine; pshenychnyi@gmail.com

**Keywords:** plasma electrolytic oxidation (PEO), ZnO nanoparticles, TiO_2_-ZnO coatings, Cp Ti grade 2

## Abstract

The paper presents the results of preliminary research on the possibility of synthesizing ZnO-TiO_2_ mixed coatings by plasma electrochemical oxidation (PEO). The aim of the work was to synthesize TiO_2_-ZnO mixed coatings on a titanium substrate from an electrolyte containing ZnO nanoparticles (NPs) and to assess the parameters of PEO on the structure, chemical composition, and properties of the obtained oxide coatings. The PEO process was carried out under various current–voltage conditions using different signals: DC, DC pulse, and AC. In this work, optimal conditions for the PEO process were determined to obtain well-adhering oxide coatings with the highest possible content of ZnO. The structure and morphology of the resulting oxide coatings were investigated, and their chemical and phase composition was comprehensively examined (EDX, XRD, XPS, and GD-OES). In addition, their basic optical properties were assessed. It has been shown that in the PEO DC pulse process, it is possible to obtain oxide coatings characterized by a high degree of structure order, high ZnO content in the oxide coating (3.6 at.%, XPS), and prospective applications for photocatalytic purposes (3.12 eV).

## 1. Introduction

ZnO thin films have been proven to be a highly desirable material in many potential applications, including solar energy systems, light-emitting diodes, photoconductive detectors, piezoelectric transducers, high-temperature superconductors, photocatalysts, etc. [1,2,3]. The interest in ZnO-based materials results from the fact that ZnO, especially in the form of nanocrystals, was recognized as a suitable alternative to titanium dioxide (TiO_2_) [4]. TiO_2_ is a semiconductor with a wide band gap of 3.2 eV (anatase) [5]. It can be activated by ultraviolet (UV) light of wavelengths below 385 nm, which greatly limits the use of sunlight as an energy source for photoreactions since only about 3–4% of the solar spectrum falls within the UV range [6]. In addition, the high recombination rate of electron/hole pairs reduces the quantum yield of a single TiO_2_ semiconductor [7]. On the other hand, despite prospects, pure ZnO-based films have a high tendency to corrode or are subject to photodegradation [8,9], which means that these materials have limited durability and therefore limited usefulness. The combination of ZnO with TiO_2_ results in the increased chemical durability of the layers [10,11]. Some researchers have proven that TiO_2_–ZnO semiconductor systems with various band gap widths can enhance the charge separation rate and visible-light photocatalytic activity compared with a single-component catalyst [12,13]. TiO_2_ is also a dielectric material and in film form presents a high resistivity. In contrast, ZnO films exhibit good conductive properties. Hence, incorporating ZnO into a TiO_2_ oxide coating can also create films with optical properties and good conductivity [14], which may be important for potential applications in photoelectrocatalysis. In several surveys over the past two decades, ZnO has been shown to possess activity against a broad spectrum of Gram-positive and Gram-negative bacteria [15,16]. This antibacterial property of TiO_2_–ZnO may therefore be of great applicational importance in the surface treatment of titanium for implantological purposes, increasing safety against post-surgical infections.

There are many fabrication techniques in the synthesis of TiO_2_-ZnO thin composite films. However, their common deposition techniques, such as sol-gel [17], spray pyrolysis [18], sputtering [19], atomic layer deposition [20], etc. (see Ref. [21]), require long reaction times and/or multi-step processing, and usually expensive chemicals and equipment. New perspectives in the synthesis of TiO_2_-ZnO composite films may be created by the plasma electrolytic oxidation (PEO) method, one of the most promising surface treatment technologies, which may be an innovative form of synthesis of thin layers based on ZnO with an oriented structure and unique physical properties. PEO is based on DC or AC polarization of a material treated at high voltage that produces plasma micro-discharges on the electrode surface, yielding high temperature (up to 5000–7000 K) and high pressure (up to 100 MPa) in its channels. As a result of local high-energy influence, layers, including both matrix (metal to be oxidized) and electrolyte elements, are formed on the product surface. In comparison to other deposition techniques, PEO is a promising technique, which is capable of fabricating ceramic coatings with a controllable porous structure at a low cost and with simple and easy operation, high metallurgical bonding strength to the substrate, and the possibility of coating formation on complex-geometry or large-size substrates [22]. Durable and mechanically stable thin films can be synthesized and easily modified by the current–voltage parameters, temperature, and chemical composition of the electrolyte. Moreover, the PEO method, due to its pro-ecological nature [23], is more advantageous in terms of environmental and economic aspects. One of the main limitations in the synthesis of coatings using the PEO method is the limited control over the local temperature increase, which may lead to significant damages in the coating in the form of cracks and non-geometric pores that are too large, which may ultimately lead to the peeling of the obtained coating from the substrate [24].

The preliminary research presented in this article proved that it is possible to obtain ZnO-TiO_2_ coatings by the PEO technique. This work is pioneering. So far, no work has been found on incorporating ZnO NPs into an oxide coating on a titanium substrate by PEO. The obtained thin films are characterized by a high degree of surface development, the homogeneous distribution of micropores, the incorporation of ZnO particles in the microstructure, and good adhesion to the titanium substrate. The experiments performed and the results obtained are a signpost for further research on the electrochemical synthesis of ZnO-TiO_2_ films and their optimization. Moreover, UV-VIS reflectance tests revealed that a band gap of the obtained films was modified in comparison to the pure TiO_2_ thin film creating a comprehensive database for the synthesis of new, long-lasting ZnO-based photocatalysts.

## 2. Materials and Methods

**Figure 1:**
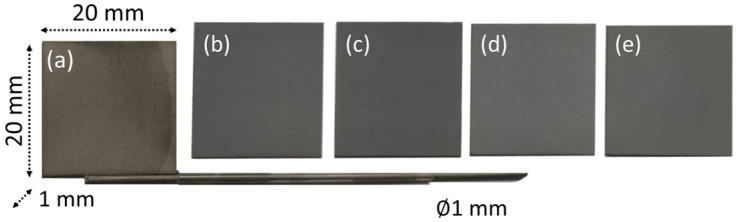
Macro photographs of the samples: after chemical etching (**a**) and after various PEO variants: DC (**b**), ZnO_DC (**c**), ZnO_DC_pulse (**d**), ZnO_AC(**e**).

The Cp titanium (Grade 2) alloy was used. The chemical composition of the alloy is shown in Table 1. The specimens were 20 mm × 20 mm × 1 mm in size and ground with SiC papers (KLINGSPOR, Schleiftechnologie, Bielsko Biała, Poland), namely PS8A of 180, 400, and 800 grits in series. The electrical connection was ensured by welding a titanium wire (Grade 2) with a diameter of 1 mm (the wire was insulated with heat shrink before the PEO process). Then, the samples were chemically etched in 10% oxalic acid solution for 1 h at boiling conditions. Next, the samples were rinsed in deionized water and forwarded to the oxidation process. Macro photographs of the samples used in this study are presented in Figure 1.

ZnO nanoparticles (NPs) were synthesized by polyol synthesis [25]. All chemicals for ZnO NPs synthesis (Zn(CH_3_COO)_2_∙2H_2_O, ethylene glycol) have been purchased from Sigma Aldrich (Darmstadt, Germany) and used as received. The crystalline size of ZnO NPs was 13.6 ± 5 nm.

**Figure 2:**
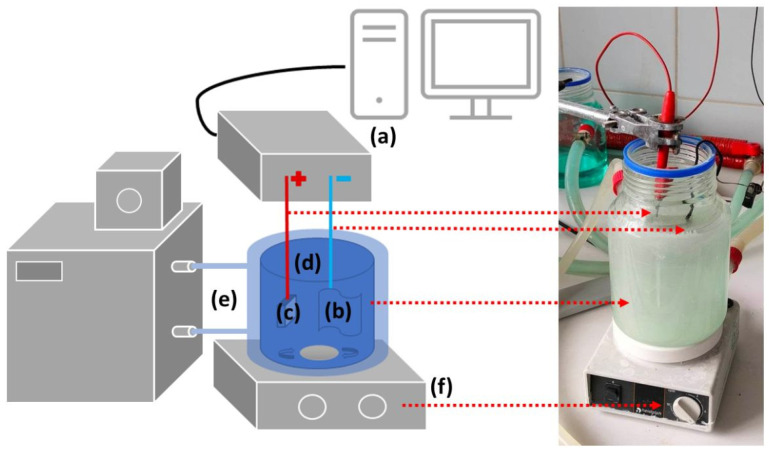
Experimental setup used for PEO along with a photo of the electrolytic cell: (**a**) power supply, (**b**) cathode, (**c**) anode (sample), (**d**) double-walled glass cell with water cooling, (**e**) thermostat, (**f**) magnetic stirrer.

The anodic oxidation was carried out in three main variants: DC, DC pulse, and AC conditions. The cathode was a titanium plate for direct current signals: DC and DC pulse and a 316L stainless steel plate for the AC signal. Before the process, the electrolyte was cooled down to 15 °C using a thermostat (Advanced Digital VWR International, Radnor, PA, USA). Two high-voltage power supplies were used during the tests: DC KIKUSUI PWR800H (Kikusui, Japan) for DC and DC pulse variants, and AC PCR1000LE (Kikusui, Japan) for the AC process. Throughout the PEO process, the electrolyte solution was stirred using a magnetic stirrer (Hei-Mix S, Heidolph, Schwabach, Germany). Figure 2 depicts a schematic diagram of the PEO experimental setup along with a photo of the electrolytic cell. The duration of the DC and DC pulse processes was 300 s, while for the AC process, 900 s. 0.05 M NaH_2_PO_2_ (NaP; Sigma Aldrich, Darmstadt, Germany) was used as the base electrolyte. For each PEO variant, 5 samples were oxidized under the same conditions. The chemical composition of the electrolyte and the anodic oxidation conditions used are summarized in Table 2.

The surface morphology of the Ti Cp alloy surfaces after PEO treatment was examined using a scanning electron microscope (SEM) equipped with an energy-dispersive X-ray analysis system (EDX, Phenom ProX, ThermoFisher Scientific, Waltham, MA, USA). The accelerating voltage was 15 kV. Additionally, the 3D reconstruction received during SEM analysis was used to determine the samples’ roughness profile (*R*_a_). The results were represented by the average of three measurements from each sample.

The X-ray photoelectron spectroscopy (XPS) analysis was conducted using an SES 2002 instrument (Scienta Omicron, Taunusstein, Germany) using a monochromatic Al K(alpha) (hν = 1486.6 eV) X-ray source (Scienta Omicron, 18.7 mA, 13.02 kV). Scan analyses were carried out with an analysis area of 1 mm × 3 mm and a pass energy of 500 eV with the energy step 0.2 eV and step time 200 ms. The binding energy of the spectrometer has been calibrated by the position of the Fermi level on a clean metallic sample. The power supplies were stable and of high accuracy. The experiments were carried out in an ultra-high vacuum system with a base pressure of about 6108 Pa. The XPS spectra were recorded in normal emission. For the XPS analyses, the CasaXPS 2.3.14 software (Shirley background type) [26] with the help of XPS tables [27,28] was used. All the binding energy values presented in that paper were charge-corrected to C 1s at 284.8 eV.

X-ray diffraction (XRD) studies were carried out using a Seifert 3003TT X-ray diffractometer (Ulm, Germany) with a copper lamp (KI1 = 1.540598 Å, KI2 = 1.544426 Å, kb = 1.39225 Å) and a nickel filter, in the angle range 2Θ 15°–80°.

The optical emission spectrometer with a glow discharge (RF GD-OES: Glow Discharge Optical Emission Spectrometer) Horiba Scientific GD Profiler 2 (Praha, Czech Republic) (was used during the tests. The Horiba Scientific GD Profiler 2 Glow Discharge Optical Emission Spectrometer (RF GD-OES). The RF GD-OES combines a glow discharge (GD) powered by an RF radio source and an optical emission spectrometer (OE). This combination allows you to quickly and accurately perform volumetric and profile analysis of the sample. Plasma conditions: pressure: 700 Pa, power: 40 W; frequency: 3000 Hz; anode diameter: 4 mm.

Photoluminescence measurements were performed according to the experimental methods, described in [29]. Briefly, photoluminescence spectra were excited with a UV laser from UltraLasers, Inc. (Toronto, ON, Canada) at an output power of 5 mW (λ = 405 nm). The photoluminescence spectrum was collected by the AvaSpec-ULS3648 fiber optic spectrometer from Avantes (Apeldoorn, The Netherlands), which recorded the signal every 15 s within 30 min of the interaction of the immunosensor with the OTA probes.

## 3. Results

### 3.1. Microstructural Characterization of PEO Coatings (SEM, EDX, 3D)

**Figure 3:**
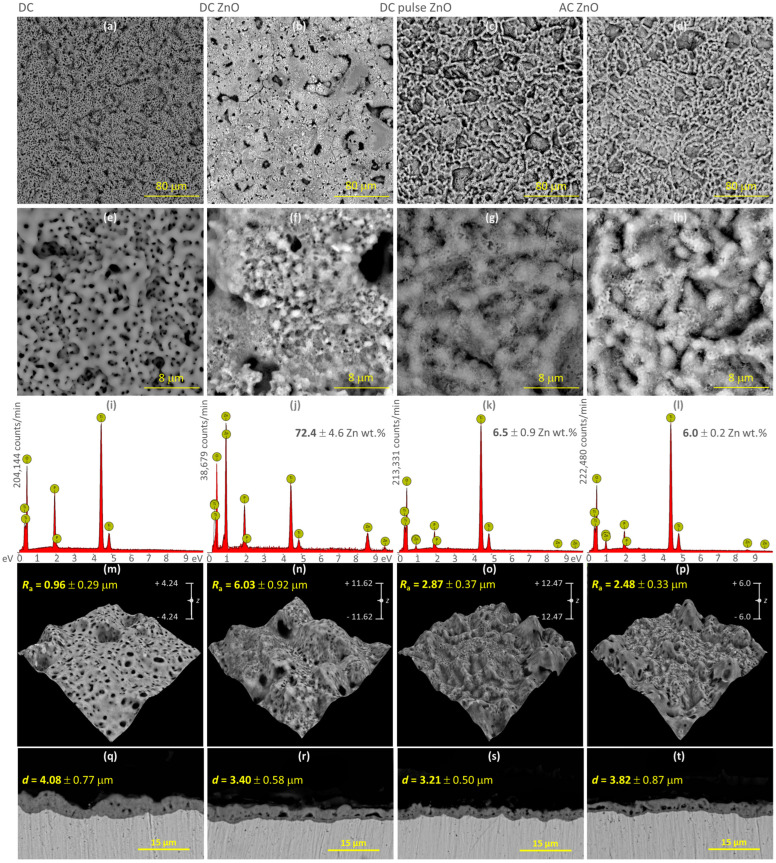
SEM images of the oxide coatings with EDX spectra*, 3D reconstruction* and cross section: (**a**,**e**,**i**,**m**,**q**) TiO_2_ coating—DC at 50 mA·cm^−2^; (**b**,**f**,**j**,**n**,**r**) ZnO−TiO_2_ coating—DC at 50 mA·cm^−2^; (**c**,**g**,**k**,**o**,**s**) ZnO−TiO_2_ coating—DCpulse at 100 mA·cm^−2^; (**d**,**h**,**l**,**p**,**t**) ZnO−TiO_2_ coating—AC 500 Hz. * average values from three measurements.

The top surface morphologies of ZnO-free and ZnO-containing coatings at 1000× and 10,000× magnification with their corresponding EDX spectra and 3D profiles can be seen in Figure 3. It was observed that the surfaces of all coatings under investigation exhibited a microporous morphology and that the pores were homogeneously distributed over the entire coating surface (Figure 3a–d). By comparing microscopic images of the samples, significant differences can be observed in the morphology of the oxide coatings. When using an electrolyte without ZnO NPs additive (Figure 3a), the oxide coating microstructure is very fine-grained and strongly surface developed. The coating was characterized by uniformity of distribution and pore size. On this basis, a high reproducibility of the oxidation process in NaP solution was found. The incorporation of ZnO NPs had a significant effect on the top surface morphology of the oxide coating, and ZnO NPs incorporation altered the main structural characteristics including porous morphology and the distribution of pores (Figure 3b–d). The coatings were characterized by fewer pores compared to the pure TiO_2_ coating. It is likely that a large part of the external pores was “blocked” by ZnO NPs agglomerates, which evenly cover the entire surface of the oxide coatings. For the DC pulse and AC conditions, similar structures were obtained in terms of their morphology (Figure 3c,d). The Zn content values given in Figure 3j–l are the average of three measurements taken at different locations on the sample surface at a magnification of x500.

The 3D SEM surface reconstructions (Figure 3m–p) were used to determine the arithmetic roughness coefficient (R_a_). Based on the R_a_, it can be concluded that the addition of ZnO NPs to the electrolyte affects the surface roughness of the oxide coating, causing a significant increase in surface development. The strongest effect was observed for the sample oxidized under DC conditions (ZnO DC). R_a_ increased to the value of 6.03 ÷ 0.92 µm, i.e., over six times higher than the roughness coefficient determined for the pure TiO_2_ oxide coating. The R_a_ values for the samples oxidized at DC pulse and AC conditions were comparable and oscillated between 2.48 ÷ 0.33 and 2.87 ÷ 0.37 µm.

As part of the research planned for this article, several variants regarding the type of current signals, current density, and/or frequency were tested. Based on the results, PEO process parameters that guaranteed the durability of the oxide coatings and repeatable results were selected. In the case of a simple DC signal used in a bath with ZnO NPs, the oxide coating often “burned”; excessive foaming of the electrolyte and an increase in the temperature of up to 70 °C were observed. The zinc content (EDX) in the final coatings was so high that the coatings tended to “fall off” the titanium substrate. Moreover, the obtained oxide coatings had visible cracks over the surface. Therefore, ZnO-TiO_2_ coating samples deposited under DC conditions were not included in further studies. Thanks to the use of a DC pulse signal, undesirable effects related to the excessive heating of the electrolyte and “burning” of the coating were eliminated. PEO tests were carried out at two current densities: 50 and 100 mA·cm^–2^. Microporous oxide coatings with stable chemical composition (EDX) and repeatable microstructure were obtained at a current density of 100 mA·cm^–2^. Thus, this DC pulse sample variant was submitted for further research. Oxidation processes using an AC signal were based on carrying out the PEO using a square wave. As part of the preliminary research for this work, several AC signal variants were tested: a frequency of 50, 500, and 999 Hz, and a duty ratio (DR) of 50:50, 30:70, and 10:90. SEM studies showed differences in the morphology of oxide coatings depending on the AC signal parameters used. It was found that as the frequency increased, the microstructure of the coatings became more fine-grained. However, it was observed that with an increase in frequency (at a given DR value), the Zn content in the oxide coating decreased. At f = 50 Hz, ZnO clusters were visible on the surface of the coatings. However, it was observed that they were unevenly distributed and locally formed adhesions that were too thick. The coatings obtained at the highest frequency of 999 Hz had characteristic, scattered, single ZnO clusters. Moreover, their microstructure was similar to the structure of the coating obtained for the DC signal, and the foaming of the electrolyte was observed during oxidation cycles. Satisfactory results were obtained using an AC frequency of 500 Hz in the PEO process. Additionally, three different DR variants were used in AC oxidation cycles. The highest Zn content in the oxide coating was obtained for the DR 50:50 variant (6.0 ÷ 0.2 Zn wt.%), for the remaining variants a decrease in the Zn content in the coating was observed: 3.2 ÷ 0.9 Zn wt.% for 30:70, and 1.4 ÷ 0.7 Zn wt.% for 10:90. Hence, samples from one AC variant, 500 Hz and DR 50:50, were submitted for further testing.

The cross-sections of the PEO oxide coatings were captured, and the thicknesses of the oxide coatings were determined from the images (Figure 3r–u; average values of coating thickness d with standard deviation are given in the images). All obtained oxide coatings were very thin, but they continuously covered the titanium substrate and were firmly attached to it. ZnO-containing samples (Figure 3q–t) showed a lower thickness in the cross-section of the oxide coating compared to the pure TiO_2_ oxide coating (Figure 3q). The thickness and internal structure of the oxide layers was found to be related to the variant of PEO process performed. The oxide coatings obtained using the DC (Figure 3r) and AC (Figure 3t) variants had numerous elongated pores in the lower part of the coating cross-section near the border with the titanium substrate. However, no structural damages in these coatings, such as transverse cracks, were found. More longitudinal pores were found for the sample oxidized under DC conditions. The most homogeneous cross-sectional structure of the TiO_2_-ZnO oxide coating was observed for the DC pulse variant (Figure 3s). Under these conditions, oxide coatings with the lowest thickness were obtained, but the internal pores were spherical, small, and evenly filled the entire cross-section of the oxide coating.

### 3.2. Chemical and Phase Composition of PEO Coatings (XPS, GD-OES, and XRD)

#### 3.2.1. X-ray Photoelectron Spectroscopy (XPS)

**Figure 4:**
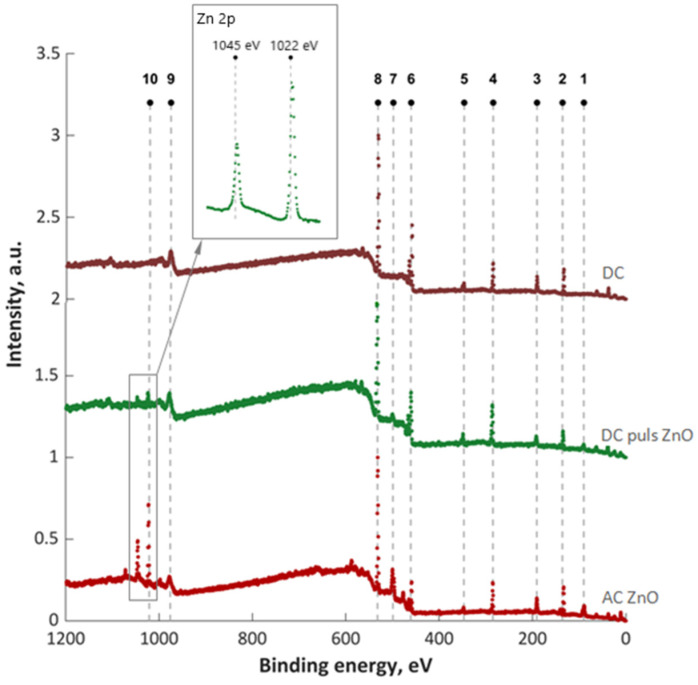
Survey spectra of the oxide coatings anodized under the selected PEO conditions. 1—Zn 3p; 2—P 2p; 3—P 2s; 4—C 1s; 5,6—Ti 2p; 7—Zn LMM; 8—O 1s; 9—O KLL; 10—Zn2p.

The XPS survey spectrum of the as-prepared oxide coatings is shown in Figure 4, and the corresponding surface elemental compositions are given in Table 3. The peak positions in all of the XPS spectra were calibrated with C 1s at 284.6 eV. The O 1s, Ti 2p, Na 1s and P 2p could be easily observed in the survey spectrum of the pure TiO_2_ oxide coating (DC). With the addition of ZnO, Zn 2p appears in the survey spectrum of ZnO/TiO_2_ oxide coatings (DC pulse ZnO and AC ZnO). Titanium was present as TiO_2_ only. It was confirmed by the doublet peaks at 458.9 and 459.0 eV [30]. Bonding energies for O 1s (530.5–531.3 eV) and P 2p (133.0–133.4 eV) indicate the presence of both oxides, phosphates, and polyphosphates.

The high-resolution XPS spectra of the binding energies at 1045 and 1022 eV in Figure 4 correspond to the Zn 2p_3/2_ and Zn 2p_1/2_ peaks. These emissions correspond to the Zn atom at the regular lattice site in ZnO [31,32]. Here, the binding energy difference between the Zn 2p_3/2_ and Zn 2p_1/2_ emissions was found to be 23 eV, which is the characteristic value for ZnO [32]. The high oxygen concentration (from 61.6 at.% up to 63.5 at.%) was recorded on the surface of each of the oxide coatings. Samples oxidized in the presence of ZnO NPs clearly differ in their Zn content. A three-times-higher atomic concentration of Zn was found on the surface of the sample oxidized under DC pulse conditions. For the same sample, a more intense incorporation of P into the oxide coating was observed (13 at.%).

**Figure 5:**
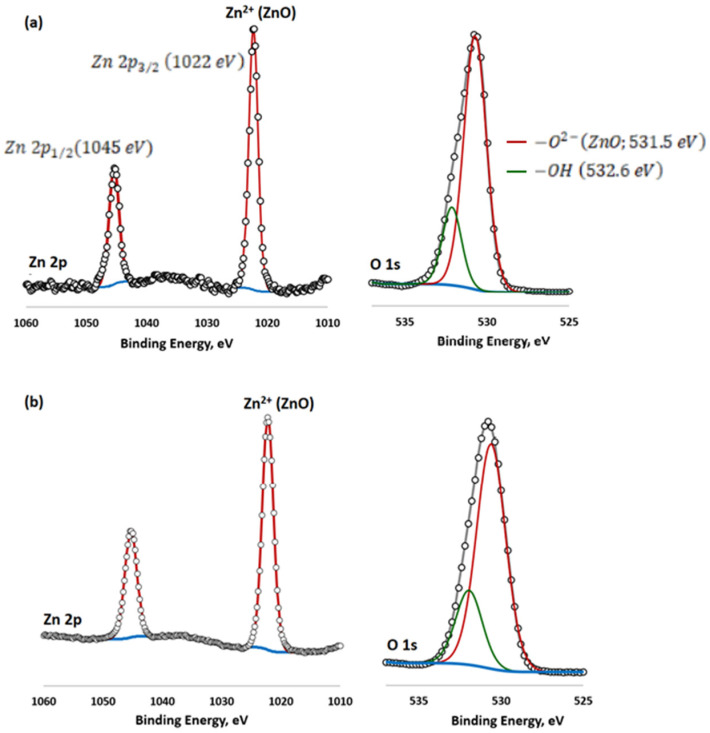
Deconvoluted spectra of Zn 2p and O1s for: (**a**) AC ZnO and (**b**) DC pulse samples, respectively. $-O^{2-}\left(ZnO;531.5\;\mathrm{eV}\right)-OH\;\left(532.6\;\mathrm{eV}\right)\;Zn\;2p_{3/2}\;\left(1022\;\mathrm{eV}\right)Zn\;2p_{1/2}(1045\;\mathrm{eV})\;$.

Analysis of the high-resolution spectra of Zn 2p an O1s (Figure 5) show that fabricated surfaces contain mainly zinc oxides mixed with titanium compounds. The deconvolution of the O 1s spectra into two peaks allows us to conclude that the first peak characterizes oxides, while the second one indicates a mixture of hydroxides, phosphates, and surface-adsorbed -OH, saturating O vacancies.

#### 3.2.2. In-Depth Composition Profile (GD-OES)

**Figure 6:**
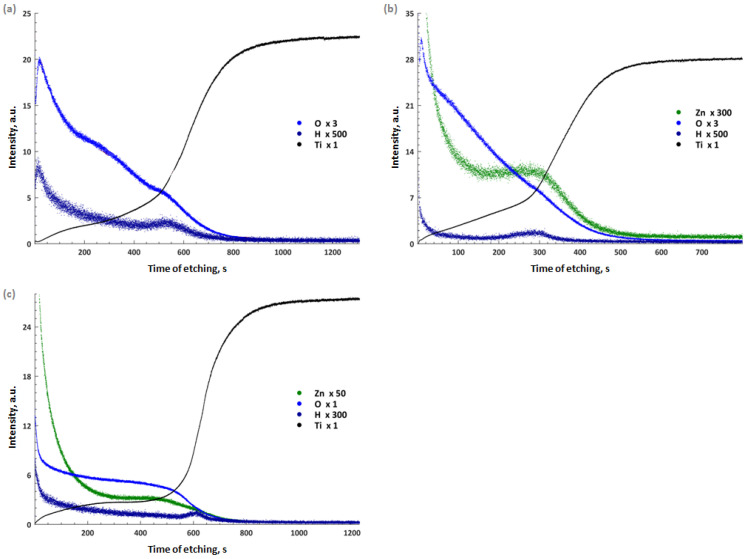
GD-OES in-depth profile of oxide coatings: (**a**) DC, (**b**) DC pulse ZnO, and (**c**) AC ZnO samples.

The in-depth GD-OES analysis of oxide coatings for selected samples and the GD-OES analysis of oxide coatings is shown in Figure 6. In the subsurface layer of the pure TiO_2_ coating (up to 50 s of etching; ~4 µm), strong signals indicating the presence of O and H were visible (Figure 6a). The surface of the Ti alloy is highly oxidized. The presence of H indicated the presence of hydrated oxides. After 50 s (~5 µm) seconds of etching, a strong decrease in the signals coming from H and O was visible, while the Ti signal began to increase. The point of intersection of the tangents drawn along the straight sections of the signal obtained for titanium (approx. 500 s; ~40 µm) was taken as the reference point where the oxide layer ends and the substrate appears.

Figure 6b,c shows the results of the profile analysis for the oxide coating containing ZnO NPs. At the beginning of etching, the strongest signals came from Zn and O. This means that the outermost nanolayer consists mainly of ZnO. The graphs also showed a slight signal coming from H with the highest intensity occurring at the beginning of etching. This indicated that the outer oxide coating was hydrated. For the DC-pulse ZnO sample, the Zn signal intensity decreased to approx. 11 V and remained constant until approx. 300 s of etching (~2.5 µm). The Zn profile line indicates the incorporation of ZnO in the entire volume of the oxide coating. The oxide layer obtained with a DC pulse signal had a lower thickness than the pure TiO_2_ coating (DC sample). Figure 6c shows the results of the GD-OES analysis obtained for the oxide coating obtained with a square AC signal. The signal of the alloy substrate (Ti) began to increase strongly after 600 s of etching (~5 µm), which indicated a higher thickness of the oxide coating formed under AC conditions compared to the DC pulse signal.

#### 3.2.3. X-ray Diffraction (XRD)

**Figure 7:**
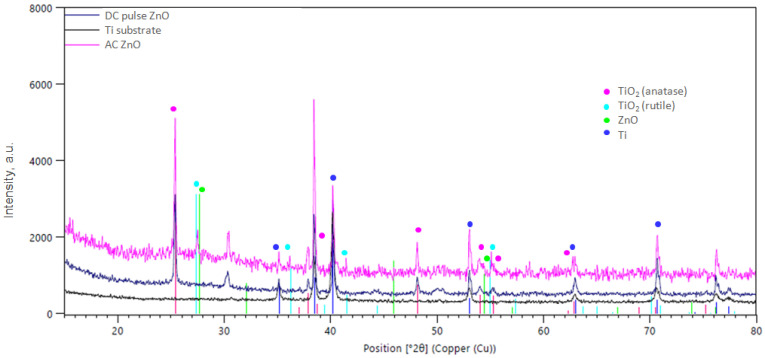
X-ray diffraction spectra for ZnO-containing oxide coatings in comparison to Ti substrate.

Comparing the XRD spectra presented in Figure 7, it was found that the main phase component of the investigated oxide coatings is TiO_2_. The overall crystalline structure shows an anatase phase with preferred (101) orientation at 2θ = 25.42° (ICDD: 04-002-8296). All other less intense diffraction peaks are assigned well to the anatase crystalline phase of TiO_2_, and another peak at 2θ = 54.53° corresponds to (211) crystal plane of the rutile phase (ICDD: 04-002-2667). The diffraction peaks of the rutile phase here are less intense than those of the anatase phase. In the case of oxide coatings obtained from an electrolyte containing ZnO NPs, peaks at 2Θ at 27.30°, 46.15°and 54.12° were observed in the XRD spectra, confirming the presence of ZnO in the oxide coating (ICDD: 04-004-4120). In the 2Θ range: 10–20°, a higher “signal lift” was also noticed, which is related to the amorphous structure of TiO_2_. No differences in phase composition were found between oxide coatings obtained in the DC pulse ZnO and AC ZnO processes.

### 3.3. Optical Properties

**Figure 8:**
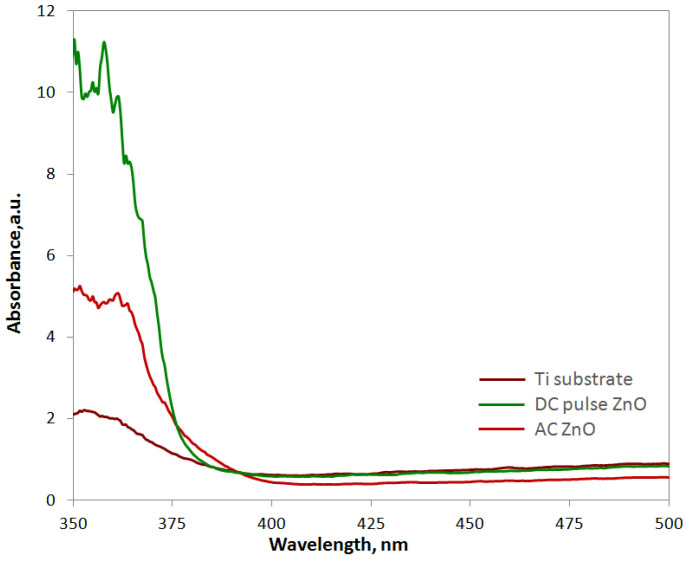
UV–Vis absorption spectra of PEO oxide coatings.

The UV–Vis absorption spectra of the investigated PEO oxide coatings are shown in Figure 8. The band gap was calculated based on the reflectance UV-Vis spectra after the Kubelka–Munk conversion using the Tauc plot method [33]. The calculated band gap values are listed in Table 4. All the oxide coatings show sharp absorption in the UV region and high transparency in the visible region. The absorption of pure TiO_2_ oxide coating (DC sample) occurred at 380 nm and increased significantly after the incorporation of ZnO on the surface of TiO_2_. In addition, the absorbance increased with the increase in Zn at.% in the oxide coating established in XPS measurements.

**Figure 9:**
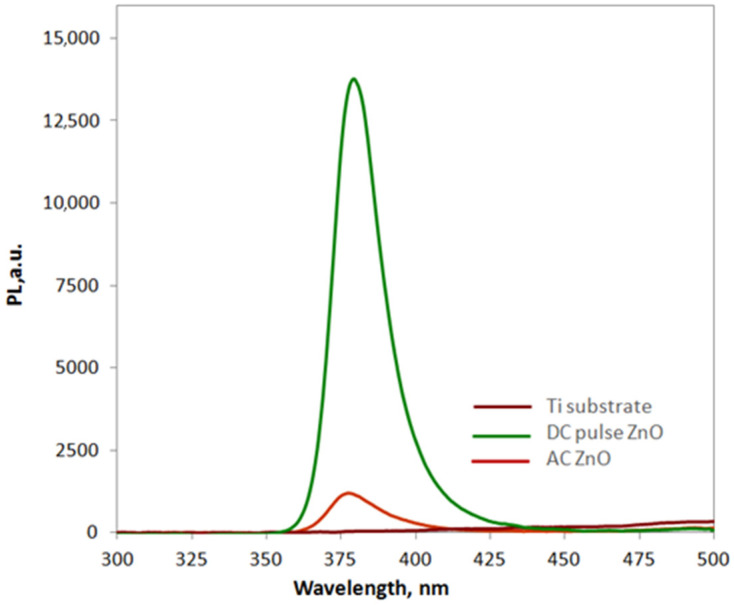
PL spectra of PEO oxide coatings.

Analyzing the values of energy gaps listed in Table 4, the lowest *E*_g_ value is characteristic of the pure TiO_2_ coating (DC sample), for samples oxidized in the presence of ZnO NPs, slightly increased *E*_g_ values were found. However, for a pure TiO_2_ oxide coating, the photoluminescence intensity at a wavelength of 380 nm was almost zero (Figure 9). Even though the ZnO-TiO_2_ coatings produced in the PEO process had a higher energy gap value, a higher photoluminescence intensity was observed for them. This effect was particularly visible for the oxide coating obtained in the DC pulse process with the highest atomic concentration of Zn in the oxide coating. It is also worth comparing the energy gap values of the obtained oxide coatings with those for pure semiconductor oxides that can be found in the literature. The hybrid material we obtained has an energy gap with a value that falls between the energy gap value of pure TiO_2_, i.e., *E*_g_ = about 3.0 eV [11], which provides activity in the UV region, and ZnO where *E*_g_ = 3.37 eV (VIS/UV_near_) [24], which is a good sign for the aspiration in obtaining a material with photocatalytic activity in visible light. In addition, they are comparable to the energy gap of other ZnO-based materials that have been studied in recent years such as Al-doped ZnO, where *E*_g_ ranges from 3.18 to 3.27 eV depending on the synthesis method used [34], or PEO-produced ZnO thin film on zinc substrate with *E*_g_ = 3.17, respectively [35].

## 4. Conclusions

The article presents the results of preliminary research on the synthesis of TiO_2_-ZnO mixed coatings, which show that it is possible to obtain durable and repeatable mixed oxide films on a titanium substrate with the sufficient incorporation of ZnO. The method of surface modification of titanium Grade 2 alloy consisting of the oxidation by PEO in an electrolyte containing ZnO NPs was presented. The influence of PEO parameters, such as current density, the type of current signal, frequency, and DR value on the structure and chemical composition of oxide coatings, was examined. Based on the obtained results, it can be concluded that each change in the parameters of the PEO process had a significant impact on the structure, chemical composition, and optical properties of the obtained oxide coatings:

1. Basic DC signal creates oxidation conditions that are too “severe” in a given electrolyte. Despite the incorporation of very high ZnO contents in the oxide coating, it does not have adequate adhesion to the titanium substrate.
2. DC pulse and AC signals enable the incorporation of ZnO into the oxide coating, maintaining the homogeneity and correctness of the porous PEO structure with the incorporation of ZnO particles in the entire volume of the oxide coating. The use of a DC pulse signal enables the incorporation of a higher ZnO content into the coating. and the structural correctness of this oxide coating cross-section (fine, round pores, and homogeneous pore distribution) indicates its best characteristics among all tested PEO variants.
3. The introduction of ZnO into the oxide coating on a titanium substrate changes its optical properties. The higher the ZnO content in the oxide coating, the higher the *E*_g_ value, but also the higher the photoluminescence intensity. Therefore, the above oxide coatings have prospective applications in photocatalytic processes. However, this requires expanded laboratory tests and verification of the results obtained in photocatalytic activity tests.

## Figures and Tables

**Table 1:** The chemical composition of Ti Grade 2 alloy.

Element	Ti	Fe	O	C	N	H	Other
wt.%	98.90	0.30	0.25	0.08	0.03	0.02	0.40

**Table 2:** The bath composition and PEO conditions.

Sample Label	Bath Composition		PEO Conditions				
	NaH_2_PO_2_ (mol·L^–1^)	ZnO (g·L^–1^)	Signal Type	Current Density (mA·cm^–2^)	Pulse Frequency (Hz)	*U*_max_ (V)	Process Duration (s)
DC	0.05	40	DC	50, 100	-	500	300
ZnO_DC			DC	50,100		500	300
ZnO_DC pulse			DC pulse	100	-	500	300
ZnO_AC			AC	-	50, 500, 999	424	900

**Table 3:** Atomic concentration fraction (%) *.

Sample Label	Na1s	P2p	O1s	Ti2p	Zn2p
DC	1.7	11.7	61.6	25.0	-
DC pulse ZnO	2.6	13.0	63.2	18.7	3.6
AC ZnO	2.6	9.8	63.5	22.9	1.2

* Average values from two measurements.

**Table 4:** The band gap values.

Sample Label	*E*_g_ (eV)
DC	2.92
DC pulse ZnO	3.12
AC ZnO	3.22

## Data Availability

Data are contained within the article.
